# Genome-Wide Association Study of QTLs Conferring Resistance to Bacterial Leaf Streak in Rice

**DOI:** 10.3390/plants10102039

**Published:** 2021-09-28

**Authors:** Xiaofang Xie, Yan Zheng, Libin Lu, Jiazheng Yuan, Jie Hu, Suhong Bu, Yanyi Lin, Yinsong Liu, Huazhong Guan, Weiren Wu

**Affiliations:** 1 College of Life Sciences, Fujian Agriculture & Forestry University, Fuzhou 350002, China; xxf317@fafu.edu.cn (X.X.); swallow1318@126.com (Y.Z.); yin21926@126.com (Y.L.); 2 Fujian Key Laboratory of Crop Breeding by Design, Fujian Agriculture & Forestry University, Fuzhou 350002, China; jackhujie1526@163.com (J.H.); bangzhu8@126.com (H.G.); 3 Fujian Academy of Agricultural Sciences, Fuzhou 350000, China; llb960119@sina.com; 4 Department of Biological and Forensic Sciences, Fayetteville State University, Fayetteville, NC 28301, USA; jyuan@uncfsu.edu; 5 College of Agriculturem, South China Agricultural University, Guangzhou 510342, China; busuhong@scau.edu.cn; 6 College of Arts College of Landscape Architecture, Fujian Agriculture & Forestry University, Fuzhou 350002, China; lins20000407@163.com

**Keywords:** rice, bacterial leaf streak, disease resistance, multi-locus GWAS, QTN

## Abstract

Bacterial leaf streak (BLS) is a devastating rice disease caused by the bacterial pathogen, *Xanthomonas oryzae* pv. *oryzicola* (*Xoc*), which can result in severe damage to rice production worldwide. Based on a total of 510 rice accessions, trialed in two seasons and using six different multi-locus GWAS methods (mrMLM, ISIS EM-BLASSO, pLARmEB, FASTmrMLM, FASTmrEMMA and pKWmEB), 79 quantitative trait nucleotides (QTNs) reflecting 69 QTLs for BLS resistance were identified (LOD > 3). The QTNs were distributed on all chromosomes, with the most distributed on chromosome 11, followed by chromosomes 1 and 5. Each QTN had an additive effect of 0.20 (cm) and explained, on average, 2.44% of the phenotypic variance, varying from 0.00–0.92 (cm) and from 0.00–9.86%, respectively. Twenty-five QTNs were detected by at least two methods. Among them, *qnBLS11.17* was detected by as many as five methods. Most of the QTNs showed a significant interaction with their environment, but no QTNs were detected in both seasons. By defining the QTL range for each QTN according to the LD half-decay distance, a total of 848 candidate genes were found for nine top QTNs. Among them, more than 10% were annotated to be related to biotic stress resistance, and five showed a significant response to *Xoc* infection. Our results could facilitate the in-depth study and marker-assisted improvement of rice resistance to BLS.

## 1. Introduction

Bacterial leaf streak (BLS) is a disease caused by the bacterial pathogen, *Xanthomonas oryzae* pv. *oryzicola* (*Xoc*), in rice. BLS is considered as one of the most destructive diseases in rice-producing regions and can cause severe rice yield loss [1]. It has been proved that the BLS resistance is quantitatively inherited in rice [2]. In general, quantitative disease resistance is controlled by multiple genes, non-race-specific and durable [3]. Therefore, the breeding of disease-resistant cultivars is a desirable approach to manage BLS in rice. To this end, it is necessary to map the quantitative trait loci (QTLs) which confer BLS resistance. Tang et al. (2000) [2] mapped eleven QTLs for BLS resistance on six chromosomes in rice. Zheng et al. (2005) [4] mapped a major QTL for BLS resistance, which could explain 13.7% of the phenotypic variance in the F2 population used. Chen et al. (2006) [5] mapped a major QTL for BLS resistance on chromosome 11. In addition, it was found that a recessive gene, *bls1*, exhibited a race-specific resistance to BLS [6], and a locus *Xo1* conferred a complete resistance to the African clade of *Xoc* strains of BLS [7]. Further, a non-host resistance gene *Rxo1* from maize displayed a qualitative resistance to BLS [8] by activating multiple defense pathways related to a hypersensitive response (HR) against *Xoc* in rice [9].

A genome-wide association study (GWAS) is a powerful alternative approach to mapping QTLs or, more precisely, quantitative trait nucleotides (QTNs). Many statistical methods are developed for GWAS, including CMLM [10], ECMLM [11], mrMLM [12], ISIS EM-BLASSO [13], pLARmEB [14], FASTmrEMMA [15], FASTmrMLM [16], and pKWmEB [17]. In these models, MLM is a mixed linear model (Q+K model); FAST is factored spectrally transformed function; ISIS is iterative-modified sure independence screening; EB is Expectation-Maximization; BLASSO is Bayesian least absolute shrinkage and selection operator; and EMMAX is an efficient mixed-model association expedited function that uses pKWmEB, pLARmEB, the Kruskal–Wallis test and the LARS algorithm, respectively. An R package called mrMLM was developed [18], in which these six multi-locus GWAS methods were integrated in the R-based software [19]. Because multi-locus GWAS models are relatively closer to the true genetic models of plants and animals than existing single-locus GWAS methods, these methods appear to display a more robust identification of QTNs with a lower false positive rate (FPR) in the analysis, especially with the small-effect quantitative trait [12,19]. Recently, GWAS is widely used to investigate the diverse and complex traits of plants, such as maize [12], barley [20], *Arabidopsis thaliana* [21] and rice [22,23,24,25].

Although several QTLs (or genes) for BLS resistance were identified in rice, they were most likely only a small part of a whole because those studies were all performed based on bi-parent crosses, from which only the QTLs (or genes) with an allelic difference between the two parents could be detected. To enhance BLS resistance breeding in rice, it is necessary to investigate a wide range of rice germplasms to map more QTLs related to BLS resistance and to identify the germplasms for better BLS resistance. A high-density single-nucleotide polymorphism (SNP) map and comprehensive HapMap were constructed in rice [26,27], and were valuable for a GWAS of complex traits. Lately, a total of seven genomic loci associated with BLS resistance were detected, based on the mixed linear model (MLM), in one season trial [28]. In our study, by phenotyping a panel of rice accessions in two seasons, we performed a multi-locus GWAS analysis of BLS resistance, aiming to identify a substantial number of loci and SNP markers related to BLS resistance, which would accelerate the genetic improvement of BLS resistance in rice.

## 2. Results

### 2.1. Population Structure and LD Pattern in the Panel

**Figure 1:**
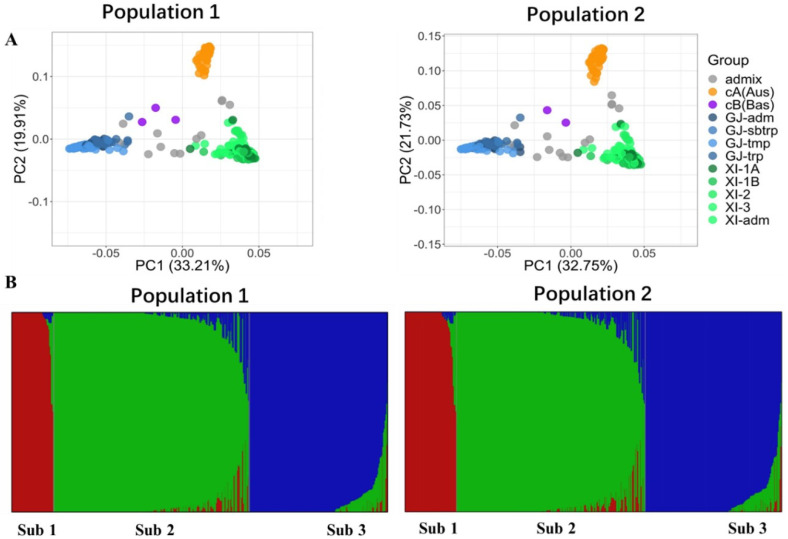
Structures of the two populations. (**A**) Population structure revealed by the plot of first two principal components. XI, indica; GJ, japonica; Aus, aus; Bas, basmati; and admix, admixed type. (**B**) Population structure is estimated by the program fastSTRUCTURE. The different colors represent subpopulations. Each vertical segment represents an accession, of which the colors indicate the degree of membership of the accession in different subpopulations.

Both PCA and fastSTRUCTURE analysis indicated that the panel of 510 rice accessions used in this study could be approximately divided into three major groups: *indica* group, *japonica* group, and circum-Aus group. In addition, there were some accessions which appeared to be genetic mixtures of the major groups. This multivariate-based partition was principally consistent with the genetic and geographic origins of the rice accessions. General speaking, this panel had a broad representation of rice (*Oryza sativa* L.). The two populations sampled from the panel for the experiments of different seasons both possessed the features of the panel, displaying very similar population structures (Figure 1).

**Figure 2:**
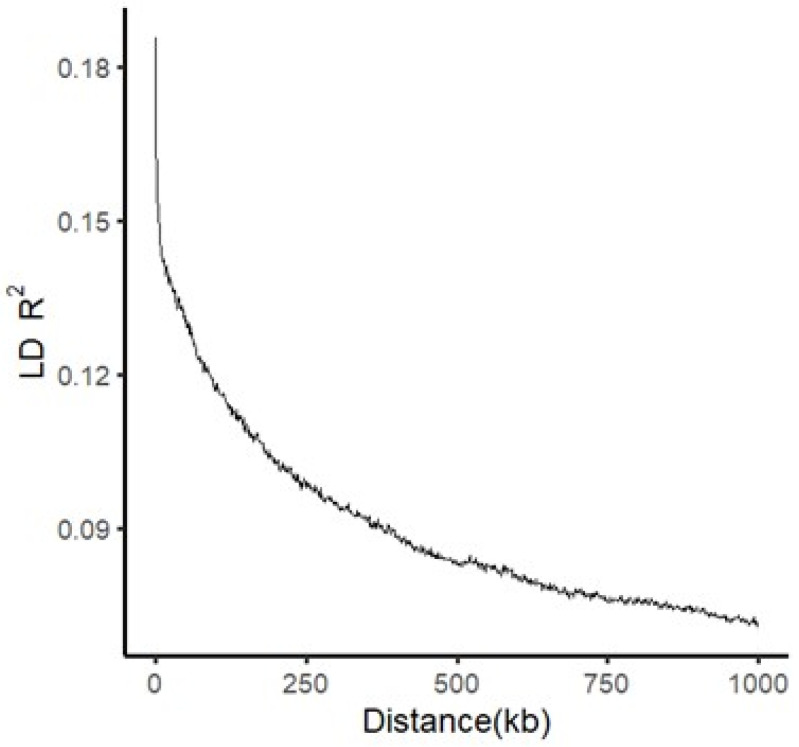
Pattern of LD decay in the whole panel of rice accessions used.

Usually, the potential of resolution in GWAS is determined by the degree of LD decay, which is influenced by several factors including population structure, allele frequency, recombination rate and selection. To evaluate the potential of resolution in this study, the relationship of LD with physical distance in the whole panel of rice accessions used was calculated. A diagram of LD (R^2^) against physical distance clearly displayed the pattern of LD decay in this population (Figure 2). The attenuation distance of LD reduction from the maximum to 0.1 and then to half of the LD values, which were ~230 kb and 330 kb, respectively.

### 2.2. Variation of Lesion Length

**Figure 3:**
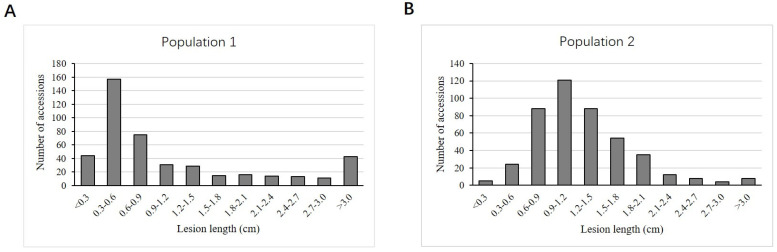
Lesion length distribution in the two populations (seasons). (**A**) Lesion length distribution of population 1. (**B**) Lesion length distribution of population 2.

The lesion length showed a wide unimodal continuous distribution skewing to the low-value direction in both populations (Figure 3). Population 1 showed a more serious skewness and larger variation (variation range = 0.15–7.83 cm, standard deviation = 1.32) than Population 2 (variation range = 0.25–5.13 cm, standard deviation = 0.59). An analysis of variance (ANOVA) of the 385 common accessions of the two populations also showed a very significant variation among the accessions (genotypes) and a very significant interaction between the genotype and environment (Table 1). However, the average variation caused by environment was not significant (Table 1). In fact, the two populations had very close mean values for BLS lesion (1.24 cm and 1.27 cm, respectively). In addition, there was a high correlation between the two populations (coefficient of correlation = 0.61, *p*-value < 0.001, calculated based on the 385 common accessions of the two populations). Moreover, the broad sense (mean-based) heritability based on the two-way ANOVA analysis for BLS lesion was analyzed using the data collected across two different environments over 2 years, and these data appeared to be quite encouraging (63.3%).

### 2.3. QTNs Associated with BLS Resistance

**Figure 4:**
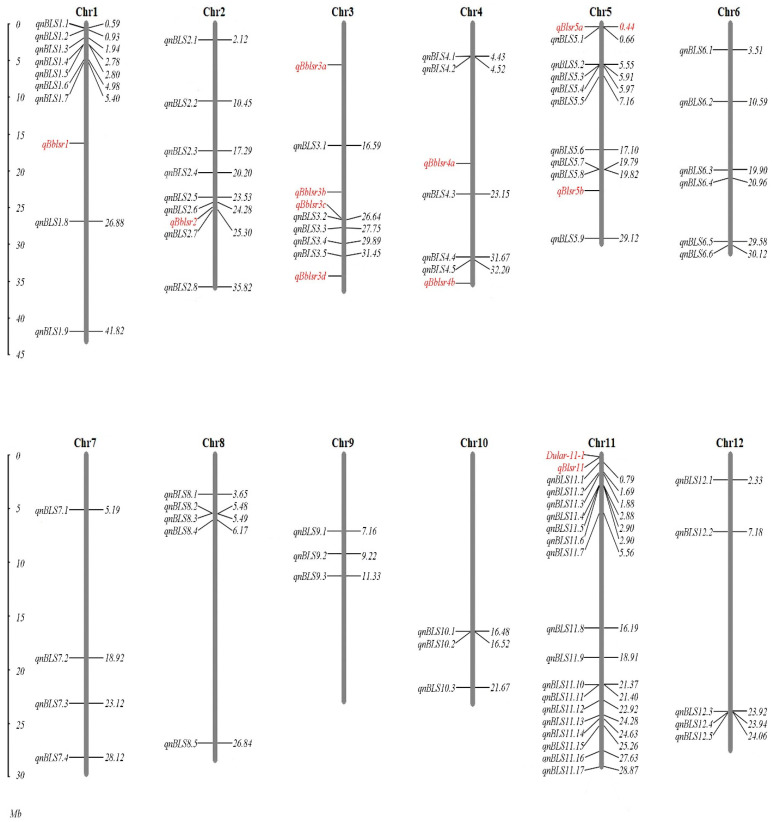
Distribution of 79 QTNs detected in this study, as well as reported QTLs/genes related to BLS resistance in the rice genome. For each chromosome, the names and physical positions (Mb) of QTNs/QTLs are shown on the left and the right, respectively. The QTNs detected in this study are shown in black, while the reported QTLs and genes are shown in red.

Using six different multi-locus GWAS methods, a total of 79 QTNs for BLS resistance were detected (LOD > 3) in the two seasons (populations), with 29 in the first season and 50 in the second season, respectively, and none were detected in both seasons (Supplementary Table S1). The QTNs were distributed on all chromosomes, with the most found on chromosome 11 (17 QTNs), followed by chromosomes 1 and 5 (9 QTNs each), and the fewest found on chromosomes 9 and 10 (3 QTNs only) (Figure 4). Most of the QTNs were detected by one method only, but 25 were detected by two or more methods simultaneously. Among them, one QTN (*qnBLS11.17*) was detected by five methods and two QTN (*qnBLS5.5* and *qnBLS12.2*) were detected by four methods (Supplementary Table S1). The additive effect of individual QTN varied from 0.00–0.92 (cm), with an average of 0.17 (cm), explaining 2.44% of the phenotypic variance, on average ranging 0.00–9.86% (Note: for a QTN detected by two or more methods, the additive effect and the percentage of the explained variance were both indicated by the mean of estimates obtained by different methods; Supplementary Table S1).

Among the 79 QTNs, 37 met at least one of the following conditions: (1) LOD > 5; (2) detected by at least two methods; and (3) an additive effect > 0.45 (Table 2). These QTNs were likely to be more reliable (based on the first and second conditions) and/or potentially more useful for breeding (based on the third condition). The statistically most significant QTN was *qnBLS8.5*, which was detected by three different methods in the first season. This QTN showed the highest values of LOD score (7.78) and the largest additive effect (0.92), explaining ~3% of the phenotypic variance (Table 2; Supplementary Table S1). In addition, ~1/3 of these 37 QTNs (including *qnBLS8.5*) displayed a significant (*P* < 0.05) interaction with the environment (Table 2).

**Figure 5:**
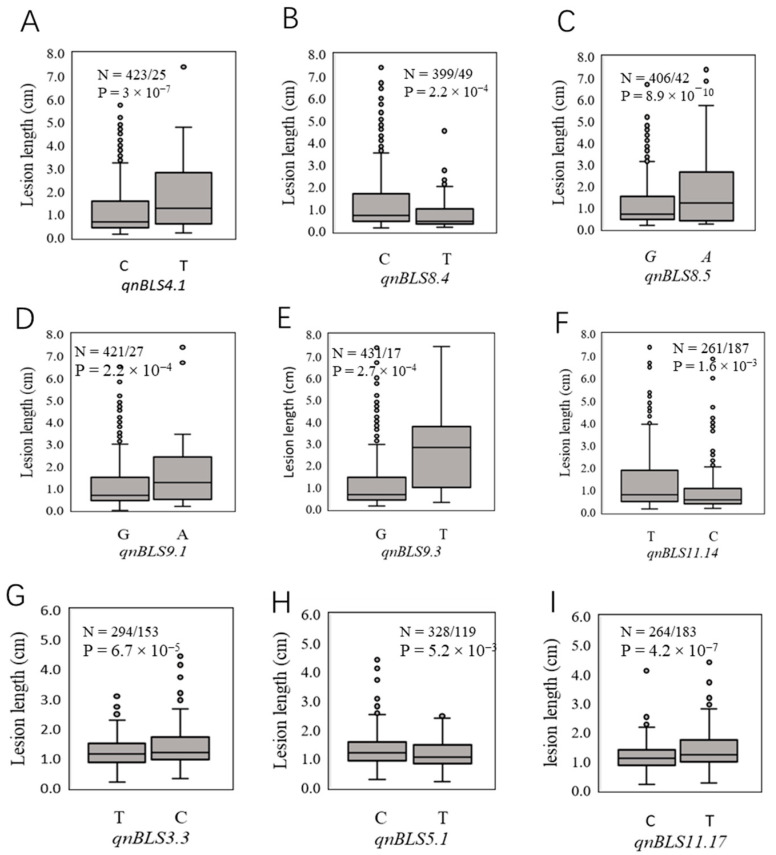
Boxplot for validating 9 selected QTNs (**A**–**I**). For each QTN, the population was divided into two groups according to allele types (N = Reference allele/SNP allele). The X-axis presented the two alleles for each QTN, while the Y-axis presented the lesion length. A t-test was used to measure the phenotypic difference of the haplotypes, and a *p*-value is shown at the top of the figure.

To evaluate the reliability of the QTNs detected, 9 top QTNs, including five which had the top LOD scores (*qnBLS8.5*, *qnBLS11.14*, *qnBLS11.17*, *qnBLS5.1* and *qnBLS3.3*) and five which had the top additive effects (*qnBLS9.3*, *qnBLS9.1*, *qnBLS4.1*, *qnBLS8.4* and *qnBLS8.5*), were individually reexamined with a t-test. The results demonstrated a significant difference in lesion length between the two genotypes of each QTN, validating the association of these QTNs with BLS resistance (Figure 5).

### 2.4. Corresponding QTLs and Candidate Genes

Based on the half attenuation distance of LD decay, the range from the upstream (330 kb) to the downstream (330 kb) of a QTN could be empirically defined as the range of QTL corresponding to the QTN. However, it is noteworthy, that there were six pairs of adjacent QTNs detected by different methods but located closely (in a distance of less than half the attenuation distance). They were: *qnBLS1.4* vs. *qnBLS1.5* (19.0 kb apart), *qnBLS5.3* vs. *qnBLS5.4* (58.4 kb apart), *qnBLS5.7* vs. *qnBLS5.8* (26.2 kb apart), *qnBLS10.1* vs. *qnBLS10.2* (39.1 kb apart), *qnBLS11.2* vs. *qnBLS11.3* (198.3 kb apart), and *qnBLS12.3* vs. *qnBLS12.4* (18.0 kb apart; Figure 4, Supplementary Table S1). In addition, although no QTN was detected in both seasons simultaneously, there were four pairs of QTNs that were detected in different seasons but located close together. They were: *qnBLS4.1* vs. *qnBLS4.2* (94.5 kb apart), *qnBLS8.2* vs. *qnBLS8.3* (12.3 kb apart), *qnBLS11.4* vs. *qnBLS11.5* (24.5 kb apart), and *qnBLS11.10* vs. *qnBLS11.11* (31.8 kb apart; Figure 4, Supplementary Table S1). Considering the influence of the experimental error and the limitation of mapping resolution, two nearby QTNs might reflect the same QTL. Therefore, the 79 QTNs detected might reflect 69 QTLs in total.

Based on the Rice Genome Annotation Project [29] annotation, the potential candidate genes of each QTL could be analyzed. There were a total of 848 annotated genes in the QTL regions of the 9 top QTNs (Supplementary Table S2). Among them, 99 putative genes were predicted to be possibly related to biotic stress resistance in function, including LRR (Leucine-rich repeat), protein kinase, zinc finger protein, PR protein, glycosyl hydrolase, MYB protein, and so on. Moreover, two predicted genes (LOC_Os09g19330 and LOC_Os11g47447) were annotated as having the bacterial resistance function of the stripe rust resistance protein Yr10.

## 3. Discussion

BLS resistance is a quantitative trait controlled by many genes in rice. So far, however, only 13 QTLs for BLS resistance are mapped in rice using the conventional QTL mapping method based on structural mapping populations derived from bi-parent cross [2,4,5]. In this study, based on a set of 510 rice varieties trialed in two seasons and using six different statistical methods, a total of 79 QTNs corresponding to 69 QTLs for BLS resistance were detected, indicating that a GWAS was much more efficient for QTL mapping than the conventional method. The main reason for this was that the conventional method only detected the genetic variation between two parents, while a GWAS detected the genetic variation among hundreds or thousands of varieties.

According to the positions of linked molecular markers, we found that the QTNs, *qnBLS2.6*, *qnBLS3.2*, *qnBLS5.1* and *qnBLS11.1*, detected in this study were very close to the reported QTLs, *qBblsr2*, *qBblsr3c*, *qBblsr5a* and *qBlsr11* [2], respectively (Figure 4; Supplementary Table S1). This suggested that the four QTNs might have reflected the four known QTLs, validating the reliability of these four QTNs. In particular, the reported QTL *qBblsr5a* was fine mapped and proved that its effect on BLS resistance was mainly, if not entirely, attributed to the known recessive gene, *xa5* [30,31]. Jiang et al. (2021) [28] presented seven BLS resistant QTNs, identified by a GWAS approach, and four of the QTNs (*qnBLS6.5*, *qnBLS6.6*, *qnBLS8.5* and *qnBLS11.7*) detected in our study appeared to be collinear with these QTNs. Our QTNs, *qnBLS6.5* and *qnBLS6.6*, were 926 kb and 387 kb, respectively, from the QTN detected in their study challenged by the pathogen strain, HNBI-19, while the QTN *qnBLS11.7* identified by two models was 146 kb from the QTN in chromosome 11. Moreover, the QTN, *qnBLS8.5*, which had the highest LOD score and was detected by three different models in our study, was only 21 kb away from the QTN obtained by the GWAS study using the same strain [28].

Most of the rice accessions trialed in the two seasons were the same in this study. However, there were many more QTNs detected in the second season than in the first season, and no common QTNs were detected in both seasons. According to the QTN positions, only four possible QTLs were commonly detected in the two seasons. These results suggested that the QTNs (QTLs) detected by GWAS were very unstable across the environments. In contrast, the conventional method usually had a much higher repeatability in QTL detection. In the study of Tang et al. (2000) [2], 6 out of 11 QTLs for BLS resistance were detected across two years. Compared with the structural populations for conventional QTL mapping, the natural populations of a GWAS usually contain many more QTLs, and the allelic frequency of a QTL often significantly deviated from 0.5. Therefore, each QTL usually had a very small heritability and was easily influenced by environmental variation in the natural populations. This implied that the QTLs detected by GWAS might have had a higher rate of false positives. This was probably a cost of the high efficiency of GWAS. Therefore, both the conventional method and GWAS had advantages and shortcomings; one could not replace the other.

The QTNs for BLS resistance were detected on all chromosomes in this study, but chromosome 11 had the largest number, many more than on any other chromosomes (Figure 4). This result appeared to be consistent with the findings that disease resistance (R) or R-like genes were enriched on chromosome 11 in rice [32,33,34,35]. It was proposed that the quantitative resistance loci (QRLs) were probably “defeated” versions of R genes with weaker phenotypes [36]. Indeed, for example, many QRLs for rice blast resistance were mapped in the same or similar locations as known R genes [24,37,38,39]. In *Brassica napus*, a whole genome study showed that 204 nucleotide binding site (NBS)-encoding genes were located in the QRLs of three diseases (blackleg, clubroot and Sclerotinia stem rot), with most in the QRLs resistant against a single disease, 20% in the QRLs resistant against two diseases, and three genes in the QRLs resistant against all the three diseases [40]. Apart from the possibility of “defeated” versions of R genes, there could be another scenario where an R gene functions as a major gene for one disease, but as a minor gene (QRL) for another disease. For instance, *xa5*, is a recessive major resistance gene against bacterial blight in rice [41,42], but it is also the most possible candidate gene of the QTL, *qBlsr5a*, conferring BLS resistance [30].

In a previous study, we identified 157 differentially expressed genes (DEGs) responding to the infection of the BLS pathogen (*Xoc*) in the rice cultivar, Nipponbare, using RNA-seq [31]. Among these DEGs, two (LOC_Os05g02060 and LOC_Os05g02070), one (LOC_Os09g12660) and two (LOC_Os11g47600 and LOC_Os11g47680) were found to be included in the lists of candidate genes for the top QTNs (QTLs); from this study, they were *qnBLS5.1*, *qnBLS9.1* and *qnBLS11.17*, respectively (Supplementary Table S2). According to the annotation, LOC_Os09g12660 encoded the glucose-1-phosphate adenylyl transferase large subunit and functioned as the chloroplast precursor, catalyzing the synthesis of the activated glycosyl donor, ADP-glucose, from Glc-1-P and ATP, which is essential for the starch synthesis of leaf chloroplasts [43]. The response of LOC_Os09g12660 to *Xoc* infection was also observed in an earlier study [9]. LOC_Os11g47600 belongs to the glycosyl transferase multigene family, which is found to be widely involved in stress responses [44,45] and related to the hypersensitive response (HR) to the pathogen, *Pseudomonas syringae* pv. *tomato* (*Pst-AvrRpm1*), in *Arabidopsis* [44,46]. LOC_Os11g47680 encodes a thaumatin family domain containing the protein (TLP), which shows elite properties in biotic and abiotic resistances [47,48,49]. The rice TLP gene exhibits an antifungal effect after it is overexpressed in banana [47]. The expression of TLP in rice also responds to a *Rhizoctonia solani* attack and environmental signals [48]. Taken together, these candidate genes potentially cause BLS resistance effects in the corresponding QTLs.

## 4. Materials and Methods

### 4.1. Mapping Panel and Field Experiments

A panel of 510 diverse rice accessions with known SNP genotypes from the public data of 3K Rice Genomes Project (2014) were used in this study (Supplementary Table S3), including 177 *japonica*, 251 *indica*, 65 circum-Aus group (cA), 3 circum-Basmati group (cB), and 14 admixed (between major groups), according to Wang et al. (2018) [27]. Two field experiments were performed in the Experimental Farm of Fujian Agriculture and Forestry, University at Yangzhong, Fujian Province (E118.485841, N26.287161), in 2019. In the first growing season (from April to August) and the second growing season (from July to October), 448 accessions (Population 1) and 447 accessions (Population 2) from the panel were planted, respectively, with 385 accessions common in the two seasons (Supplementary Table S3). In both seasons, rice seeds were sown after pre-germination and 10 seedlings per accession were transplanted 25 d after sowing in the paddy field, with a distance of 20 cm between rows and between seedlings. Field management followed the regular procedure.

### 4.2. BLS Resistance Assessment and Broad Sense Heritability

Pathogen inoculation was conducted at the active tillering stage of rice with a highly virulent *Xoc* strain kindly provided by Prof. Guoying Cheng of Huazhong Agricultural University, using the pricking inoculation approach [2,30]. Five leaves with similar age were inoculated in each plant and three of them were randomly selected for measuring the longitudinal length of lesion on the leaf with a ruler 18 d after inoculation. The average lesion length of three leaves was calculated for each plant, and the average lesion length of 5 plants was calculated to indicate the BLS resistance of each accession. The broad sense (mean-based) heritability analysis from two-way ANOVA was conducted using the following equation: h^2^ = σG^2^/[σG^2^ + (σGE^2^/e) + (σe^2^/re)] where σG^2^ (genetic variance), σGE^2^ (genotype-environment interaction variance) and σe^2^ (variance of error) were measured and normalized using e (number of environment) and r (number of replicates) [50]. R [51] was used in the statistical analysis including two-way ANOVA and broad sense heritability with its native packages. The significant level of the assessed traits was displayed using R package car (type II Wald chi-square tests) [51].

### 4.3. Multi-Locus GWAS

The SNP data of 510 rice accessions were obtained from the 3K Rice Genomes Project database [52]. A subset of 140345 SNPs meeting the requirement of minor allele frequency (MAF) 5% and missing data ratio < 0.1 were selected from the core genome set of 404K SNPs [53] for population and association analyses. The population structure was investigated using the method of PCA (principal component analysis) plots and the program fastSTRUCTURE [54] (http://rajanil.github.io/fastStructure/). LD decay for the whole population was measured by correlation coefficients (R2) for all pairs of SNPs within 1000 Kb using the tool of plink [55].

The mrMLM (Multi-Locus Random-SNP-Effect Mixed Linear Model) package was downloaded from database [18]. Six multi-locus GWAS methods implemented in the mrMLM package, including (1) mrMLM [12], (2) FASTmrEMMA [15], (3) pLARmEB [14], (4) ISIS_EM-BLASSO [13], (5) pKWmEB [17] and (6) FASTmrMLM [16], were used to analyze the data, respectively. The kinship matrix (K matrix) was calculated according to the SNP information following Xu et al. (2013) [56]. An empirical LOD score threshold of 3 was set to identify associated QTNs as suggested [12,57].

### 4.4. Comparison of Phenotypic Differences Corresponding to QTNs and Identification of Candidate Genes

The phenotypic value for each accession was collected based on the SNP information as that of reference allele and target allele, respectively. A t-test was conducted to assess the phenotypic differences between the two groups. To obtain reliable candidate genes, the QTNs with the top five LOD scores and top five values of additive effect were annotated based on the Rice Genome Annotation Project (http://rice.plantbiology.msu.edu/). The interval of half LD attenuation distance from the upstream to the downstream of a QTN was empirically defined as the region of the corresponding QTL.

## 5. Conclusions

With six different multi-locus GWAS methods (mrMLM, ISIS EM-BLASSO, pLARmEB, FASTmrMLM, FASTmrEMMA and pKWmEB), a total of 79 QTNs for BLS resistance were identified, these QTNs were not evenly distributed on rice genome. Each QTN had an additive effect of 0.20 (cm) on average and explained the cumulative degree of phenotypic variation. The novel QTNs identified in this study can be used to improve rice variety resistance to BLS through marker-assisted study.

## Figures and Tables

**Table 1:** Two-way analyses of variance of lesion lengths of 385 accessions planted in two seasons.

Source of Variation	df	SS	MS	F
Genotype (G)	384	2737.919	7.130	36.104 ***
Environment (E)	1	0.504	0.504	2.553
G × E	383	1020.639	2.665	13.494 ***

*** indicates the significance at *p* ≤ 0.001.

**Table 2:** Details of 37 important QTNs associated with BLS resistance in rice.

QTN	Chr.^a^	Pos. (bp) ^b^	ARG ^c^	Season	LOD ^d^	Effect ^e^	PVE ^f^	MAF ^g^	Method ^h^	*p*-Value ^i^
*qnBLS1.1*	1	592,207	A	1	6.11	0.31	2.89	0.46	**3**	0.12
*qnBLS1.4*	1	2,778,821	A	1	7.02	0.25	3.07	0.35	**6**	0.14
*qnBLS1.8*	1	26,876,161	T	2	7.13	0.18	8.47	0.47	1,5,**6**	0.29
*qnBLS2.4*	2	20,195,417	T	1	5.87	0.32	3.98	0.45	**3**,6	0.06
*qnBLS2.5*	2	23,526,303	A	1	5.31	−0.30	3.05	0.29	**1**,3	0.01
*qnBLS2.6*	2	24,279,697	A	1	6.26	−0.32	5.14	0.21	**5**	0.00
*qnBLS2.7*	2	25,304,627	T	2	7.14	−0.11	1.81	0.21	1,**3**,6	0.10
*qnBLS3.2*	3	26,640,636	A	1	5.94	0.29	0.98	0.12	**3**,6	0.00
*qnBLS3.3*	3	27,746,259	C	2	7.27	0.11	4.97	0.42	**1**,3	0.77
*qnBLS4.1*	4	4,426,267	T	1	3.11	0.61	1.22	0.07	**2**	0.31
*qnBLS5.1*	5	655,785	T	2	7.41	−0.15	4.76	0.29	1,**5**	0.49
*qnBLS5.3*	5	5,913,003	A	1	4.83	0.29	2.56	0.23	1,**3**	0.00
*qnBLS5.5*	5	7,156,565	T	2	5.62	0.09	1.94	0.24	1,3,**5**,6	0.16
*qnBLS5.7*	5	19,789,079	A	2	4.99	0.11	2.33	0.23	5,**6**	0.61
*qnBLS5.8*	5	19,815,239	G	2	5.28	−0.05	0.54	0.47	**3**	0.43
*qnBLS6.2*	6	10,585,154	A	2	3.70	−0.01	1.11	0.27	3,**5**	0.74
*qnBLS6.4*	6	20,963,067	A	2	5.34	0.17	1.81	0.11	2,**5**	0.34
*qnBLS6.5*	6	29,582,089	C	1	5.42	−0.46	9.86	0.47	**1**	0.00
*qnBLS8.1*	8	3,647,393	A	2	6.60	−0.07	1.73	0.39	**5**	0.89
*qnBLS8.2*	8	5,476,860	A	1	5.50	0.18	0.82	0.27	**3**,6	0.02
*qnBLS8.4*	8	6,165,793	T	1	3.20	−0.60	2.25	0.15	**2**	0.02
*qnBLS8.5*	8	26,839,948	A	1	7.78	0.59	3.49	0.10	1,**2**,6	0.00
*qnBLS9.1*	9	7,158,144	A	1	5.99	0.92	2.88	0.07	**2**	0.00
*qnBLS9.3*	9	11,334,496	T	1	4.20	0.75	1.66	0.07	**2**	0.05
*qnBLS11.3*	11	1,879,265	T	1	5.41	−0.39	7.49	0.40	**1**	0.08
*qnBLS11.4*	11	2,876,450	A	1	4.95	−0.52	2.95	0.27	**2**	0.14
*qnBLS11.6*	11	2,901,282	G	2	3.35	0.14	2.00	0.13	**1**,3	0.39
*qnBLS11.7*	11	5,561,289	A	2	3.62	0.08	0.41	0.08	**5**,6	0.55
*qnBLS11.11*	11	21,402,757	A	1	3.40	0.18	1.04	0.26	3,**6**	0.06
*qnBLS11.13*	11	24,281,128	A	2	4.40	0.05	1.00	0.19	1,**3**,6	0.08
*qnBLS11.14*	11	24,628,136	C	1	7.68	0.20	2.43	0.45	**3**	0.05
*qnBLS11.17*	11	28,874,298	T	2	7.55	0.14	4.61	0.44	1,**3**,4,5,6	0.35
*qnBLS12.1*	12	2,330,084	C	1	6.54	0.31	4.34	0.50	3,**6**	0.01
*qnBLS12.2*	12	7,182,082	C	2	7.01	0.23	3.72	0.15	1,2,**4**,5	0.26
*qnBLS12.3*	12	23,920,185	C	2	4.87	0.09	1.66	0.21	**2**,5	0.31
*qnBLS12.4*	12	23,938,101	G	2	5.17	0.13	4.10	0.37	3,**6**	0.31
*qnBLS12.5*	12	24,055,853	G	1	5.20	0.32	4.11	0.23	**1**,5	0.01

Note: a. Chromosome. b. Position. c. Allele in the reference genome. d. The maximum LOD score obtained by the methods. e. Mean additive effect estimated by the methods. The negative sign indicates that the allele in the reference genome acted to decrease the trait value (lesion length). f. Mean percentage of phenotypic variance explained estimated by the methods. g. Minor allele frequency. h. 1: mrMLM, 2: FASTmrEMMA, 3: pLARmEB, 4: ISIS_EM-BLASSO, 5: pKWmEB, 6: FASTmrMLM. The bold number indicates the method that obtained the highest LOD score. i. *p*-value of interaction with environment.

## Supplementary Materials

The following are available online at https://www.mdpi.com/article/10.3390/plants10102039/s1, Table S1: Details of loci associated with BLS via multi-locus GWAS in rice. Table S2: Candidate gene under QTNs. Table S3: Rice accessions trialed in two seasons.
